# Supplementary material for: Assessing the Expression of Long INterspersed Elements (LINEs) via Long-Read Sequencing in Diverse Human Tissues and Cell Lines
Source: Genes (Basel). 2023 Sep 29;14(10):1893. doi: 10.3390/genes14101893 (PMC10606529; doi:10.3390/genes14101893)
Supplement: Supplementary file 1 [file genes-14-01893-s001.zip › TE_Supplementary_Materials_proof.pdf]

SUPPLEMENTARY MATERIALS

SUPPLEMENTARY TABLES

Table S1. Sample Reference, Sample Preparation Method, Sequencing Library Protocol, and Sequencing Platform. This table presents information related to samples used in the study, along with their associated preparation protocols and sequencing platforms. The "Sample Reference" column includes reference numbers from Gene Expression Omnibus (GEO), BioProject, or the Singapore Nanopore Expression (SG-NEx) project, as well as other relevant references. The "Sample Preparation Method" column describes the specific methods used to prepare the samples before sequencing. The "Library Construction Protocol" column provides details about the protocols used when constructing the sequencing libraries and the "Sequencing Platform" column indicates the type of platform used from ONT or PacBio for sequencing.

Table S2. LongReadsum Mapping Statistics Initially and Following RepeatMasker, Mapping to hg38 Reference Genome, and Artifact-Based Filtering. This table presents the sample name, initial number of reads, N50 read length, and the median read length obtained from sequencing, number of reads over 1kb in length, the number of reads remaining after RepeatMasker, total number of reads successfully mapped to the hg38 reference genome, N50 mapped read length, median mapped read length, total number of supplementary alignments, and total number of reads following the artifact-based filtering portion of the methods.

Table S3. Average Number of Reads, scaled to reads per million, for the Active, Intact ORF2-Only, and Inactive L1 Elements across Tissue and Cell Line Samples. This table presents the average L1 expression values for three different categories of LINE-1 elements: active, inactive, and intact only in ORF2 L1s. The values were normalized by the total number of reads in the sample following mapping to the hg38 reference genome, and scaled to reads per million.

Table S4. Manually Reviewed Unique L1 Loci Genomic Locations with Corresponding Average Number of Reads, scaled to reads per million, for the Active L1 Elements across the PacBio sequenced Cell Lines. This table presents the genomic locations, unique identifier (UID), L1 subfamily (L1HS or L1PA2), and the average L1 expression values, scaled to reads per million, of the 40 unique active L1 loci with expression across the PacBio sequenced cell lines, including: ESCC KYSE140, ESCC KYSE510, ESCC TE5 (samples 1 and 2), ESCC SHEEC, ESCC SHEE (normal), HepG2, K562 cell lines, and UHR.

SUPPLEMENTARY FIGURES

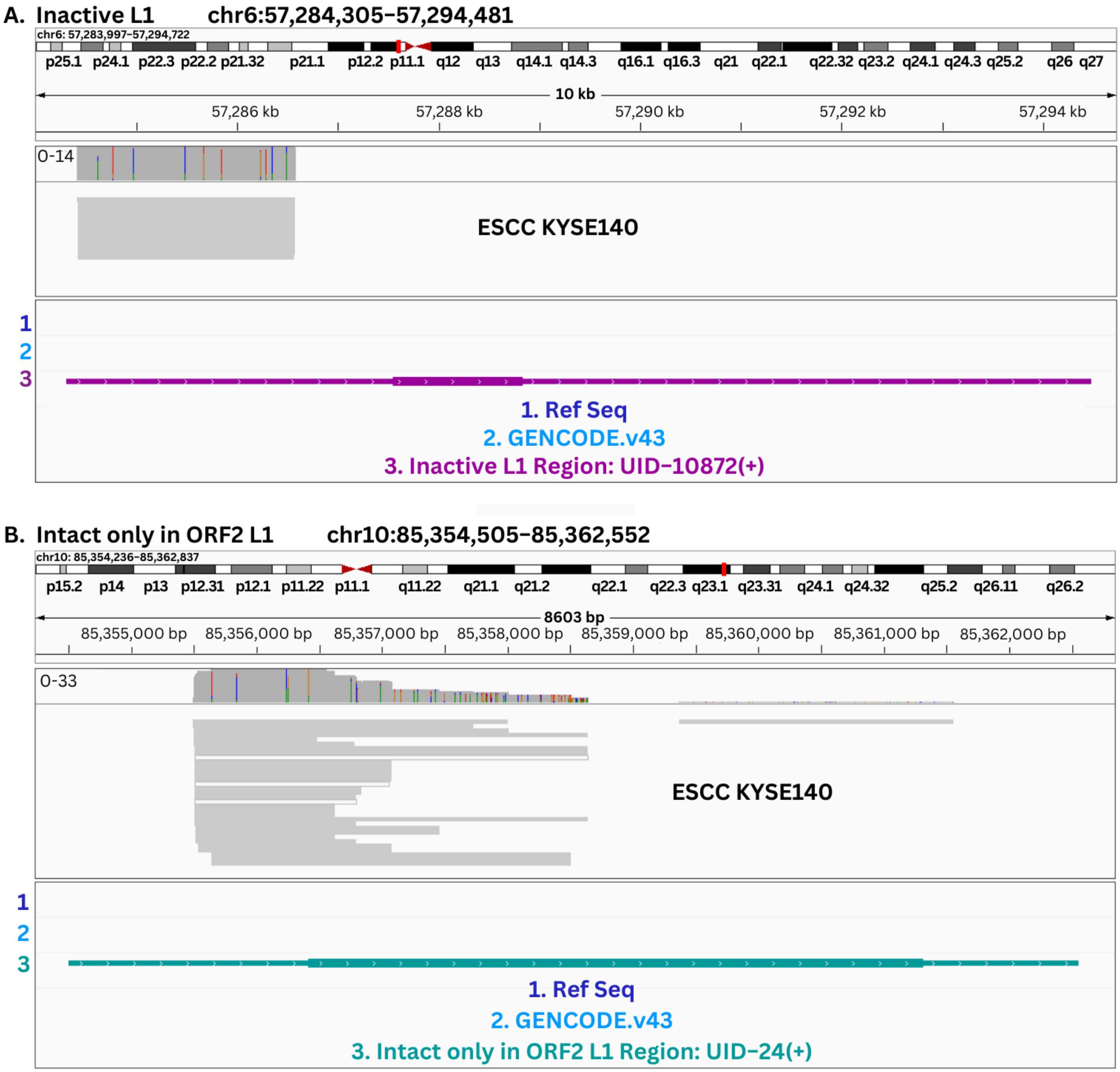

Figure S1. Manual validation of inactive and intact only in ORF2 L1 regions within ESCC KYSE140 cancer cell line using the Integrative Genomics Viewer (IGV). (A) Expression of an inactive L1 region (chr6:57,284,305-57,294,481, hg38) from one cancerous ESCC KYSE140 sample. **(B)** Expression of an intact only in ORF2 L1 region (chr10:85,354,505-85,362,552, hg38) from the same cancerous ESCC KYSE140 sample. The bottom panel shows reference annotations including, RefSeq (hg38, dark blue), GENCODE.v43 [45] (light blue) with the respective strandness, and the inactive L1 region (purple) or the intact only in ORF2 L1 region (teal) from the L1Base2 reference with the respective strandness and unique identifier (UID) of the L1 element. For each sample, the sequencing reads are represented by grey lines, with the coverage showing in the top section.

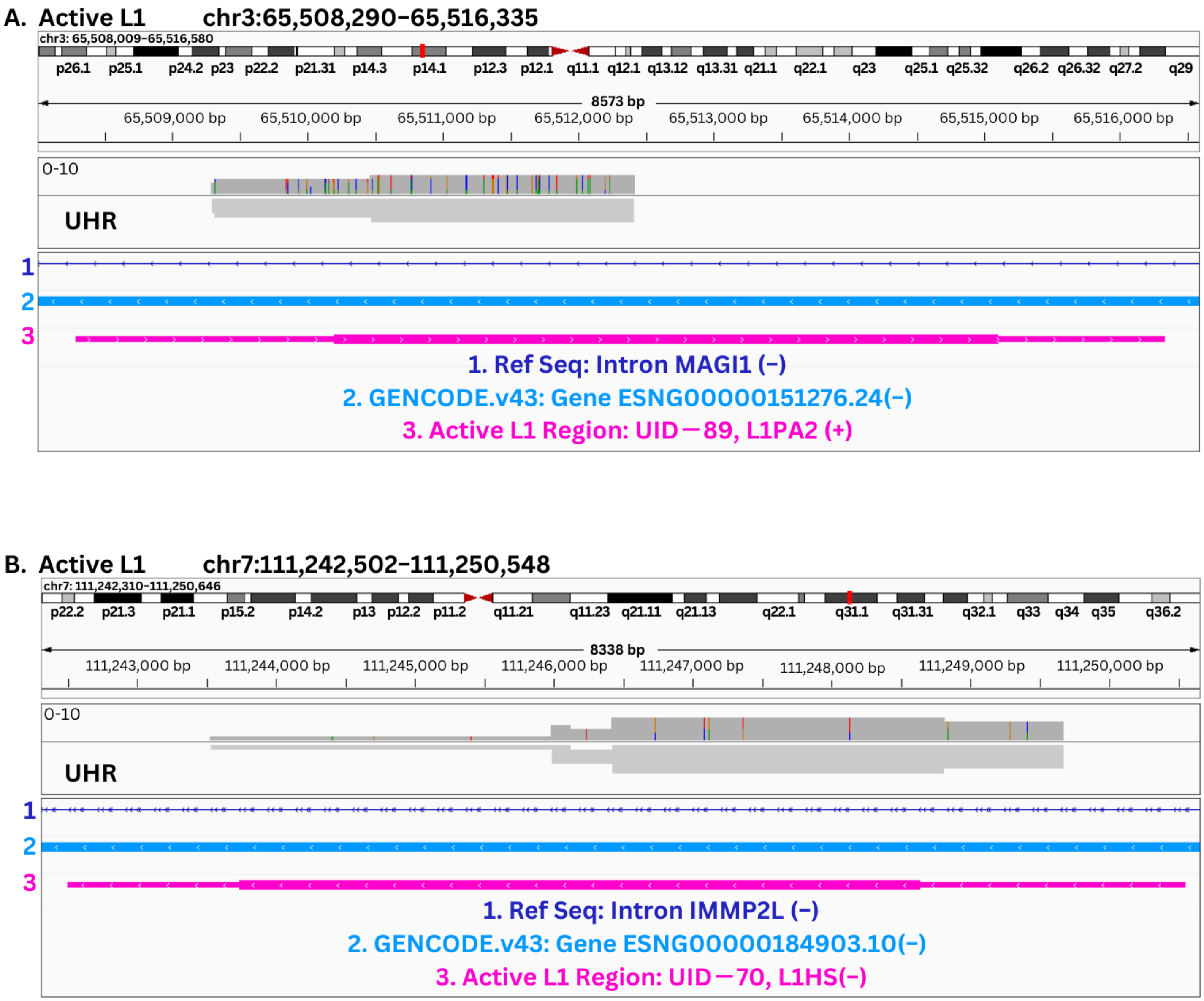

Figure S2. Manual validation of inactive and intact only in ORF2 L1 regions with high coverage within the UHR cancer cell line, sequenced by PacBio, using the Integrative Genomics Viewer (IGV). Expression of **(A)** an active L1 region (chr3:65,508,290-65,516,335, hg38) and **(B)** an active L1 region (chr7:111,242,502-111,250,548, hg38) from UHR cell line sample. The bottom panel shows reference annotations including, RefSeq (hg38, dark blue), GENCODE.v43 [45] (light blue) with the respective strandness, and the active L1 regions (pink) from the L1Base2 reference with the respective strandness, unique identifier (UID), and subfamily name of the L1 element. For each sample, the sequencing reads are represented by grey lines, with the coverage showing in the top section.

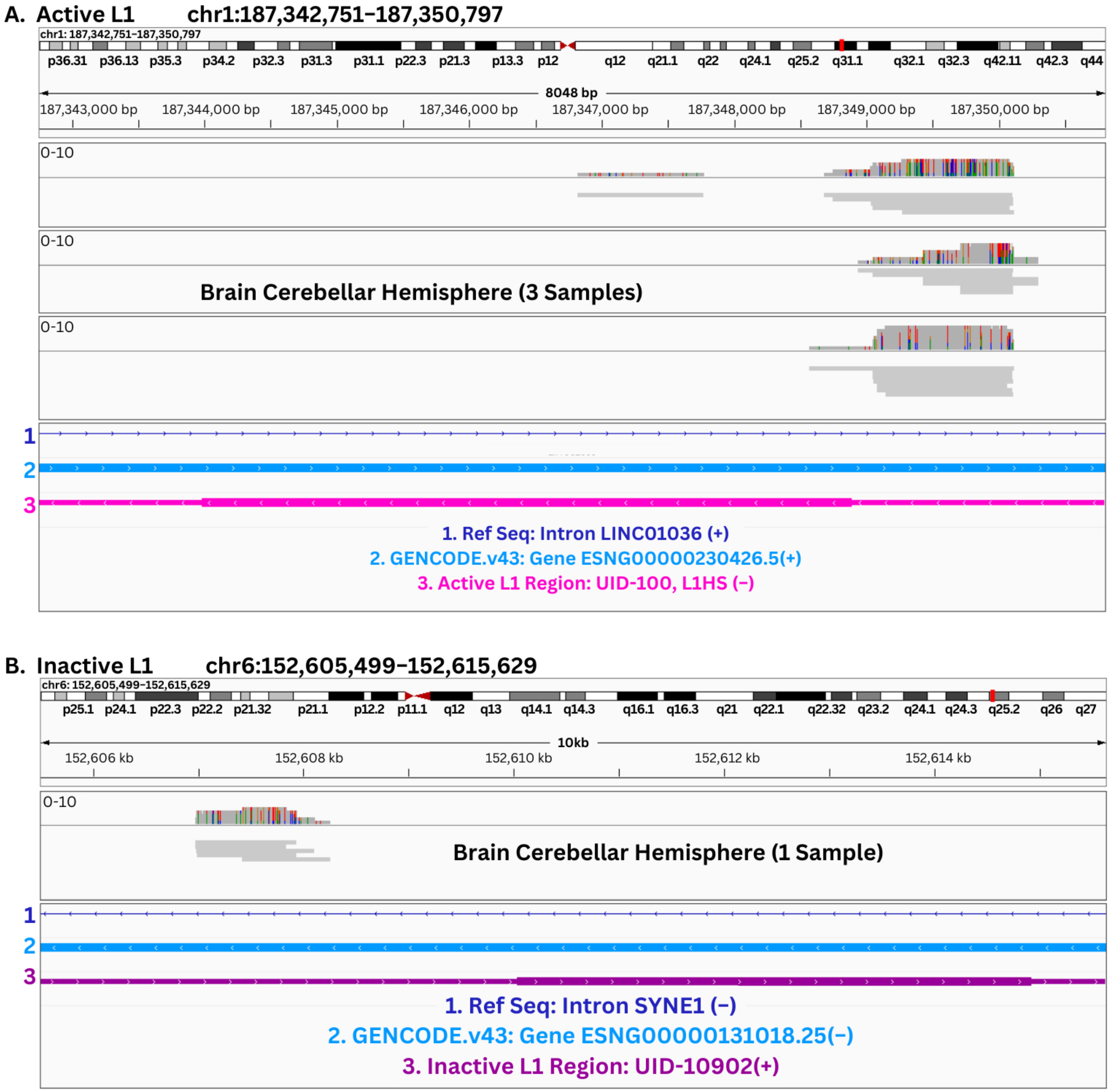

Figure S3. Manual validation of active and inactive L1 regions with high coverage within human brain tissues from the GTEx project using the Integrative Genomics Viewer (IGV). (A) Expression of the shared active L1 region (chr1:187,342,751-187,350,797, hg38) among three human cerebellar hemisphere brain tissue samples. **(B)** Expression of the shared inactive L1 region (chr6:152,605,499-152,615,629, hg38) from one human cerebellar hemisphere brain tissue sample. The bottom panel shows reference annotations including, RefSeq (hg38, dark blue), GENCODE.v43 [45] (light blue) with the respective strandness, and the active L1 region (pink) or the inactive L1 region (purple) from the L1Base2 reference with the respective strandness, unique identifier (UID), and/or subfamily name of the L1 element. For each sample, the sequencing reads are represented by grey lines, with the coverage showing in the top section.
